# Profound hypotony maculopathy in a first episode of bilateral idiopathic acute anterior uveitis

**DOI:** 10.1186/s12886-015-0105-0

**Published:** 2015-08-28

**Authors:** Matthew R. Edmunds, Simon N. Madge

**Affiliations:** Academic Unit of Ophthalmology, University of Birmingham, Birmingham & Midland Eye Centre, Dudley Road, Birmingham, B18 7QH UK; Hereford County Hospital, Stonebow Road, Hereford, HR1 2BN UK

**Keywords:** Anterior Uveitis, Hypotony, Hypotony Maculopathy

## Abstract

**Background:**

We report a case of a HLA-B27 negative patient presenting with severe, bilateral, idiopathic acute anterior uveitis with acute hypotony and hypotony maculopathy as their first uveitic episode.

**Case presentation:**

Within a week of onset of her first episode of acute anterior uveitis, a 45 year-old Caucasian lady developed profound ocular hypotony with unrecordable intraocular pressures, reduced vision and choroidal folds. All investigations were negative. Uveitic hypotony responded slowly to corticosteroids – intravenous, oral and topical – with normalization of intraocular pressure and resolution of choroidal folds after two months. Anterior uveitis and hypotony have not returned with six months of follow-up.

**Conclusion:**

Bilateral, profound hypotony maculopathy may present acutely in idiopathic acute anterior uveitis, may be slow to respond to treatment and should be considered as a cause of vision loss in patients with this condition.

## Background

Ocular hypotony is a known complication of trauma, chronic retinal detachment, vitreoretinal or glaucoma filtration surgery (particularly with the use of Mitomycin-C), as well as uveitis [1]. Maculopathy associated with hypotony is important as it is associated with reduced visual acuity in a high proportion of patients and may be challenging to treat [2, 3]. Previous cases of hypotony in the context of HLA-B27 have been reported, likely due to ciliary body inflammation with reduced aqueous production, or increased uveoscleral outflow [4, 5]. We report an unusual case of bilateral profound, prolonged hypotony with maculopathy in the context of a patient’s first episode of idiopathic acute anterior uveitis.

## Case presentation

A 45-year-old Caucasian lady, with no significant past ocular or medical history, presented with bilateral ocular pain, redness and photophobia. Visual acuities at first attendance were 6/9 OU. She had evidence of 3+ anterior chamber cells and 2+ flare bilaterally with no fibrin, hypopyon or posterior synechiae. There was no vitritis or retinitis. Initial intraocular pressures (IOP) were 10 mmHg OU by Goldmann applanation tonometry (GAT). There were no systemic symptoms, no predisposing factors for uveitis and no personal or family history of HLA-B27-related disease. The patient was commenced on regular guttae Dexamethasone 0.1 % six times per day and Cyclopentolate 1 % twice daily.

**Fig. 1:**
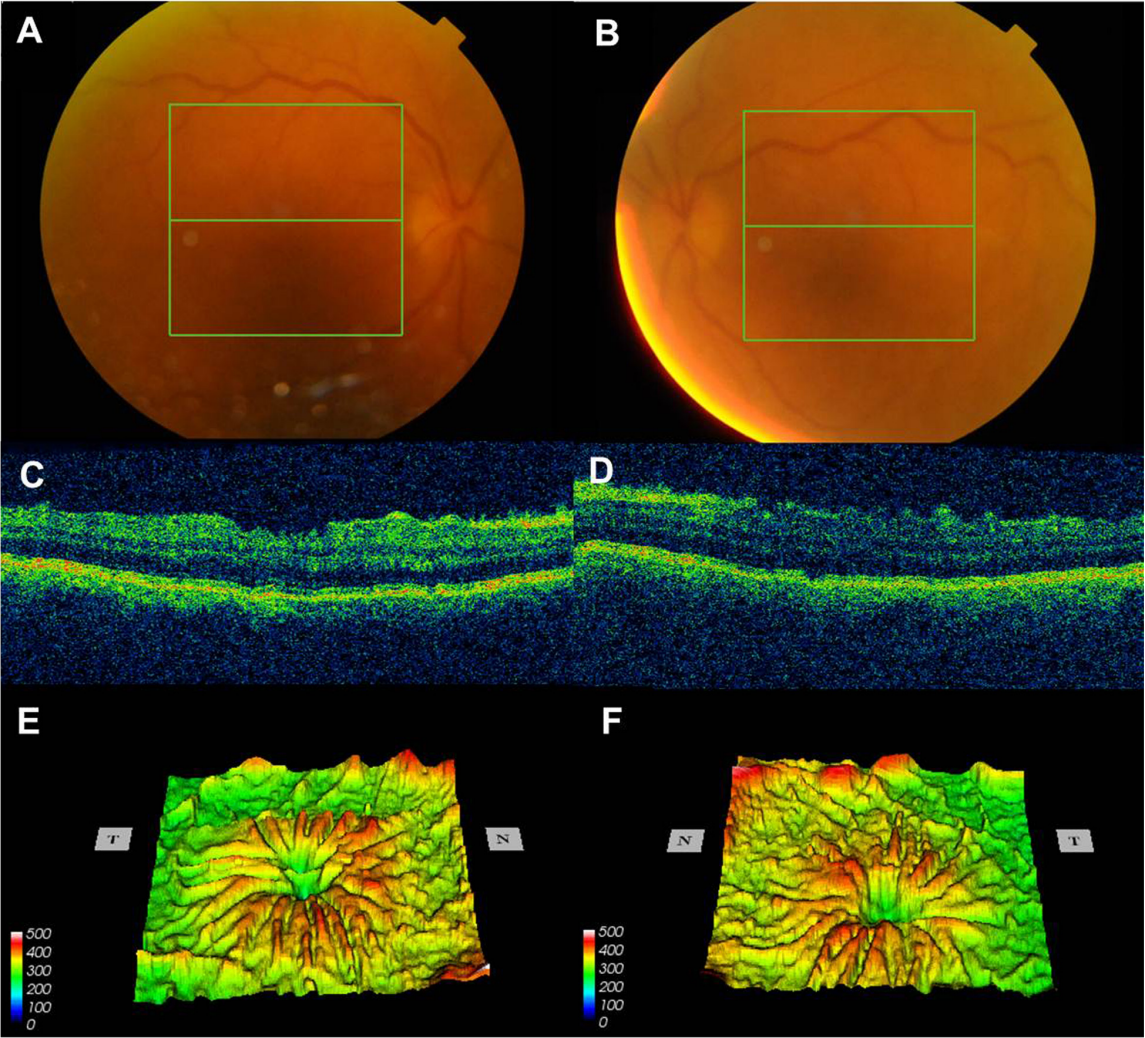
Color fundus photographs of right (**a**) and left (**b**) eyes demonstrating poor hazy view, principally due to Descemet’s membrane folds. There was no evidence of vitreous activity. Optical coherence tomography images of the right (**c**) and left (**d**) macula with irregular retinal contour, further demonstrated on OCT thickness map of the right (**e**) and left (**f**) macula

On review one week later visual acuities had deteriorated to hand movements at one metre, bilaterally. Anterior chamber activity was unchanged but intraocular pressures were now unrecordable and there were bilateral Descemet’s membrane folds. Fundal examination revealed bilateral choroidal folds (Fig. 1a and b), with optical coherence tomography confirming no cystoid macular edema or epiretinal membrane (Fig. 1c and d). There was no evidence of optic disc edema, peripheral choroidal detachment or exudative retinal detachment. Gonioscopy revealed open iridocorneal angles.

A diagnosis was made of hypotony maculopathy secondary to acute anterior uveitis with iridocyclitis. She was commenced on guttae Prednisolone 1 % hourly and Atropine 1 % twice daily and received intravenous methylprednisolone 1 g on three consecutive days, before continuing with oral Prednisolone 60 mg, reducing gradually over the next several months.

All investigations were unremarkable, with normal full blood count, urea and electrolytes, c-reactive protein and erythrocyte sedimentation rate, angiotensin converting enzyme, anti-nuclear antibodies, anti-neutrophil cytoplasmic antibodies, immunoglobulins, complement C3 and C4 and protein electrophoresis. Treponemal and Bartonella serology were negative, as was that for HIV. HLA-B27 was negative. Chest X-ray demonstrated no abnormality. No aqueous sample or vitreous biopsy was taken.

Hypotony continued for the next two months, with IOP measured at 2 – 4 mmHg bilaterally and visual acuities varying between hand movements and 6/24, but eventually stabilized with IOP of 13 mmHg on each side. With resolution of hypotony maculopathy visual acuities returned to 6/6 OU. The patient’s vision remains stable with no further episodes of anterior uveitis or hypotony, six months after resolution of the episode.

## Discussion

To our knowledge this is the first report of a HLA-B27 negative patient presenting with acute hypotony in their first uveitic episode. Chronic uveitic hypotony is more common than acute hypotony. While chronic cases are thought to develop due to prolonged inflammatory activity, with ciliary body atrophy or the development of cyclitic membranes, acute hypotony in uveitis has been proposed to be due to ciliary body inflammation, with reduced aqueous production, or increased uveoscleral outflow [2, 3].

Both acute and chronic hypotony may result in hypotony maculopathy, optic nerve edema, exudative retinal detachment and choroidal folds, although acute cases are more likely to be reversible and less likely to be associated with phthisis bulbi [2]. Nevertheless, a high proportion of patients with uveitic hypotony have significantly reduced vision, with one cohort determining that 84.1 % of eyes presenting with hypotony had a visual acuity of 20/200 or worse [3].

The published prevalence of hypotony related to uveitis varies depending on the patient cohort. In the Multicenter Uveitis Steroid Treatment (MUST) Trial, in which hypotony was defined as an eye with IOP of <7 mmHg at the baseline visit or an eye with a history of hypotony and an IOP of <9 mmHg at the baseline visit, 8.3 % of 240 uveitic patients had hypotony. Mean IOP in hypotonous eyes in the MUST Trial was 4.6 mmHg and hypotony was bilateral in only three cases (1.25 %) [2].

However, only 1.86 % of 6785 patients in a non-infectious uveitis cohort from the Systemic Immunosuppressive Therapy for Eye Diseases (SITE) cohort developed hypotony, equivalent to an incidence of 0.006 per person-year. In this cohort hypotony was defined as IOP <5 mmHg sustained for ≥2 visits spanning at least 30 days. Of 11,119 eyes in 6545 patients uveitic patients, 189 eyes (1.7 %) of 161 patients presented with hypotony (IOP <5 mmHg), with 28 (17.4 % of the hypotonous patients, 0.4 % of the total patients) presenting with bilateral hypotony [3]. It is uncertain how many of these patients were presenting with their first episode of uveitis as some were commented to have band keratopathy.

In these cohorts hypotony was found to be more common in patients with greater duration of uveitis (≥5 years) or with a history of previous ocular surgery (e.g. cataract surgery, pars plana vitrectomy). Furthermore, those of African-American ethnic origin, with hypertension or diabetes mellitus and uveitis from a younger age had more hypotony. Hypotony was also more common in anterior than intermediate or posterior uveitis and in those with more severe uveitis (e.g. anterior chamber flare of +1 or more, posterior synechiae and pars planitis) [2, 3]. In the study of Daniel et al. [3], patients with panuveitis had the highest prevalence of hypotony (4.8 %), followed by anterior uveitis (1.8 %), posterior uveitis (0.6 %) and intermediate uveitis (0.3 %). Rates of hypotony in pediatric uveitis, particularly JIA-associated uveitis (often an anterior uveitis) are higher [6].

Hypotony maculopathy has previously been described in patients with anterior uveitis associated with HLA-B27, but our patient was HLA-B27 negative. Of the six HLA-B27 positive patients in the literature with uveitic hypotony, only one had bilateral anterior uveitis and hypotony [4, 5]. This was a 61-year-old male with left hypopyon, right fibrinous uveitis and bilateral vitritis, seemingly a much more severe uveitic case than the patient we present [5].

## Conclusion

To our knowledge this is the first report in the literature of a HLA-B27 negative patient presenting with their first episode of bilateral idiopathic acute anterior uveitis characterized by profound hypotony and hypotony maculopathy, and who responded slowly but completely to topical and systemic corticosteroid. Ophthalmologists will be aware of hypotony in the context of chronic or recurrent uveitis, but should also be aware of such significant and debilitating hypotony in those previously naïve to intraocular inflammation.

## Consent

Written informed consent was obtained from the patient for publication of this Case report and any accompanying images. A copy of the written consent is available for review by the Editor of this journal.

## Abbreviations

GAT: Goldmann applanation tonometry
HIV: Human immunodeficiency virus
HLA-B27: Human leucocyte antigen B27
IOP: Intraocular pressure
mmHg: Millimetres of mercury
MUST: Multicenter Uveitis Steroid Treatment
OU: Oculus uterque (both eyes)
SITE: Systemic Immunosuppressive Therapy for Eye Diseases

## References

[1] Fannin LA, Schiffman JC, Budenz DL (2003). Risk factors for hypotony maculopathy. Ophthalmology.

[2] Sen HN, Drye LT, Goldstein DA, Larson TA, Merrill PT, Pavan PR, Sheppard JD, Burke A, Srivastava SK, Jabs DA; Multicenter Uveitis Steroid Treatment (MUST) Trial Research Group. Hypotony in patients with uveitis: the Multicenter Uveitis Steroid Treatment (MUST) Trial. Ocul Immunol Inflamm. 2012;20:104–12. 10.3109/09273948.2011.647228 PMC3610172 22409563

[3] Daniel E, Pistilli M, Pujari SS, Kaçmaz RO, Nussenblatt RB, Rosenbaum JT, Suhler EB, Thorne JE, Foster CS, Jabs DA, Levy-Clarke GA, Kempen JH (2012). Risk of hypotony in noninfectious uveitis. Ophthalmology.

[4] Roe R, Branco BC, Cunningham ET (2008). Hypotony maculopathy in a patient with HLA-B27-associated uveitis. Ocul Immunol Inflamm.

[5] van der Veer EG, Keunen JE, Rothova A (2014). Severe HLA B27-associated uveitis complicated by hypotony, serous retinal detachment, and ciliochoroidal effusion. Ocul Immunol Inflamm.

[6] Thorne JE, Woreta F, Kedhar SR, Dunn JP, Jabs DA (2007). Juvenile idiopathic arthritis-associated uveitis: incidence of ocular complications and visual acuity loss. Am J Ophthalmol.

